# Reply to de la Barra Ortiz, H.A.; Chamorro Lange, C.H. Comment on “Alayat et al. The Effectiveness of Photobiomodulation Therapy on Pain and Function in Patients with Patellofemoral Pain Syndrome—A Systematic Review and Meta-Analysis. *J. Clin. Med.* 2026, *15*, 20”

**DOI:** 10.3390/jcm15197735

**Published:** 2026-10-08

**Authors:** Mohamed Salaheldien Alayat, Roaa A. Sroge, Abdulaziz Awali, Ammar Fadil, Omair Belal Malibari, Raad Hatim Ajawi, Eyad Noor Wali, Suhail Hafiz, Sameer Yamani

**Affiliations:** 1 Medical Rehabilitation Sciences Department, College of Applied Medical Sciences, Umm Al-Qura University, Mecca 21955, Saudi Arabia; rasroge@uqu.edu.sa (R.A.S.); amawali@uqu.edu.sa (A.A.); asfadil@uqu.edu.sa (A.F.); pt.omair.malibari@hotmail.com (O.B.M.); raadajawi@gmail.com (R.H.A.); eyadnoor.w@gmail.com (E.N.W.); suhailhaviz@gmail.com (S.H.); 2 Basic Science Department, Faculty of Physical Therapy, Cairo University, Giza 12613, Egypt; 3 Physical Therapy Department, Umm Al-Qura University Medical Center, Mecca 21955, Saudi Arabia; syamani@uqu.edu.sa

Authors would like to sincerely thank the authors of the commentary [1] for their careful reading of our published systematic review and meta-analysis and for providing valuable methodological and statistical insights. Their observations highlight important considerations in evidence synthesis and contribute positively to improving transparency and interpretation in future systematic reviews on photobiomodulation (PBM) therapy. Below, authors provide a detailed point-by-point response to each concern raised.

## 1. Handling of Multi-Arm Trials and Unit-of-Analysis Error

The commentators noted that some trials with multiple arms may have contributed more than one comparison to the same meta-analysis [2,3], potentially introducing unit-of-analysis errors.

Authors appreciate this important methodological observation. In our review, multi-arm studies were included because they provided relevant PBM comparisons against different active modalities [2,3]. However, authors acknowledge that including more than one comparison from the same study in a pairwise meta-analysis can artificially increase the weight of that study and violate the independence assumption. The Cochrane Handbook recommends several strategies such as combining arms, selecting one comparison, or splitting the shared control group [4,5].

In our analysis, RevMan automatically treated each comparison separately, which may have led to correlated comparisons. No additional sensitivity analysis or post hoc check was performed to quantify the effect of these correlated comparisons on the pooled estimates; therefore, the magnitude of any resulting bias remains uncertain. Authors agree that a more conservative approach (splitting the shared control group or combining PBM arms) could strengthen the robustness of pooled estimates. Authors acknowledge this as a methodological limitation and recommend that future meta-analyses apply Cochrane-recommended adjustments or consider network meta-analysis approaches when multiple active comparators exist [4,5,6,7].

## 2. Heterogeneity of Co-Interventions in “PBM + Exercise” Group

The authors stated that the subgroup “PBM plus exercise vs. corresponding controls” included heterogeneous co-interventions (manual therapy, taping, electrotherapy), which makes it difficult to isolate PBM effects.

Authors fully agree with this point. Although the label “PBM + exercise” provides a simplified grouping for meta-analysis purposes, clinical protocols varied substantially across included trials. Some studies combined PBM with additional interventions such as manual therapy, soft tissue mobilization, electrotherapy, and multimodal physiotherapy programs.

Authors acknowledge that these co-interventions may exert independent effects on pain and function and may confound the isolated contribution of PBM. However, in most sham-controlled trials, PBM was the only independent variable that differed between groups, which strengthens the inference that the observed effect is attributable primarily to PBM. Nevertheless, variability in rehabilitation content remains a source of clinical heterogeneity and may partly explain the high I^2^ values observed [4,5].

The limited number of studies investigating the effect of PBM therapy on PFPS may be the main cause for NOT applying a subgroup analysis that considered each intervention with its corresponding control. Authors agree that more focused subgroup analyses (PBM + exercise vs. sham PBM + exercise only) could provide more clinically interpretable evidence. This is an important recommendation for future work, especially if more RCTs become available.

## 3. Use of SMD Instead of Mean Difference (MD) for Pain Outcomes

The commentators suggested that since pain was mainly measured using VAS, pooling using mean difference (MD) would be more clinically interpretable than standardized mean difference (SMD).

Authors appreciate this valuable methodological suggestion. Authors started the meta-analysis with MD for pain since it was measured by a 10-cm scale using different methods of evaluation such as visual analogue scale (VAS) and numerical pain rating scale (NPRS). The initial review by the reviewer suggested performing a new meta-analysis using SMD rather than MD since the measurement scales were differ.

Our decision to use SMD was based on the presence of different pain rating scales across trials (VAS and NPRS/NRS), which required standardization to allow pooling. Although VAS was the dominant tool, at least one trial reported pain using NPRS. Therefore, SMD was chosen to ensure compatibility across all included comparisons.

However, authors agree that when all studies use a uniform scale (e.g., VAS 0–10), MD is preferable because it provides results directly interpretable in clinical units. Additionally, MD-based pooling may reduce unnecessary heterogeneity and improve interpretability for clinicians [7,8].

Authors acknowledge that reporting pooled results as MD (VAS points) or presenting back-transformed SMD estimates would improve clinical understanding. This issue is especially relevant because the minimal important difference (MID) used in GRADE was defined in original scale units [7,8,9]. Therefore, Authors agree that back-transformation could strengthen the alignment between pooled estimates and MID interpretation.

Authors recognize this as an area for improvement in future updates of the review.

## 4. Inconsistency in Reporting VAS vs. NRS and Clinical Non-Equivalence

The comment highlighted an inconsistency in reporting VAS in all trials, while the text mentioned that one study used NRS/NPRS. They also raised concerns that VAS and NRS are correlated but not interchangeable.

Authors thank the commentators for identifying this reporting inconsistency. Authors confirm that one included study assessed pain using NPRS/NRS rather than VAS, and the extracted data should have reflected this distinction. This represents a reporting oversight, and authors agree that clearer reporting is required to avoid confusion. 

Authors also agree with the methodological argument that correlation does not guarantee equivalence. Although VAS and NRS measure pain intensity and are often pooled together, they differ in measurement properties (continuous vs. ordinal), scaling, and agreement [10,11]. This supports the rationale for our choice of SMD rather than MD. However, authors acknowledge that this approach reduces clinical interpretability.

Authors appreciate this important clarification and agree that future analyses should explicitly describe the scale used in each trial and consider sensitivity analysis excluding studies using different instruments.

## 5. Pooling of Functional Outcomes (AKPS and WOMAC) and Directional Issues

The authors noted that AKPS and WOMAC assess different constructs and may not be appropriate to pool together. They also highlighted possible directional inconsistencies in one trial [12].

Authors agree that AKPS and WOMAC are distinct functional instruments and pooling them may introduce indirectness and additional heterogeneity [13,14]. In our review, authors attempted to harmonize outcome direction by transforming WOMAC scores (multiplying by −1), so that higher scores reflected functional improvement consistently across instruments.

**Figure 1:**
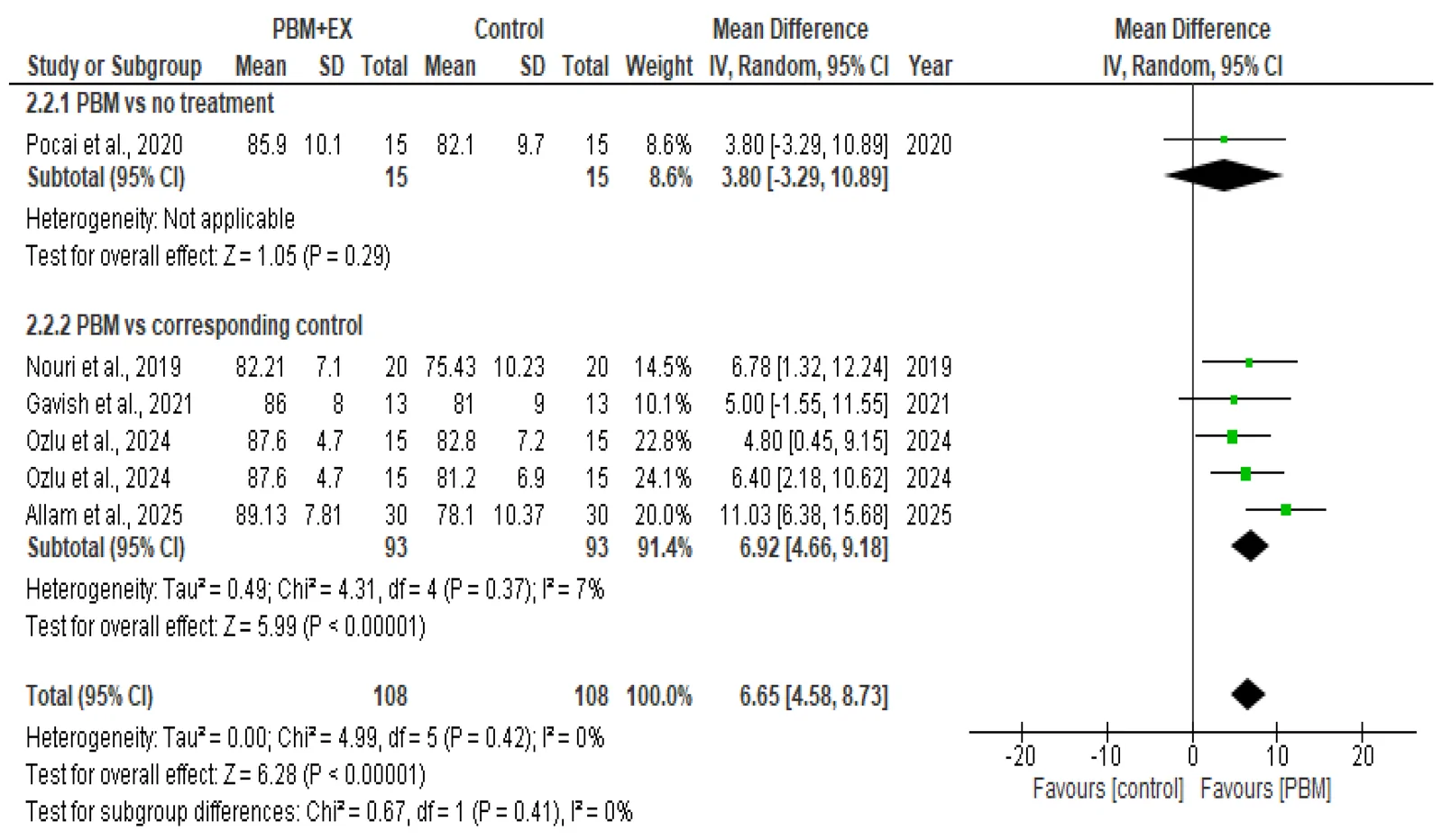
Forest plot of effectiveness of PBM on function (Higher score is better results) [2,3,15,16,17].

**Figure 2:**
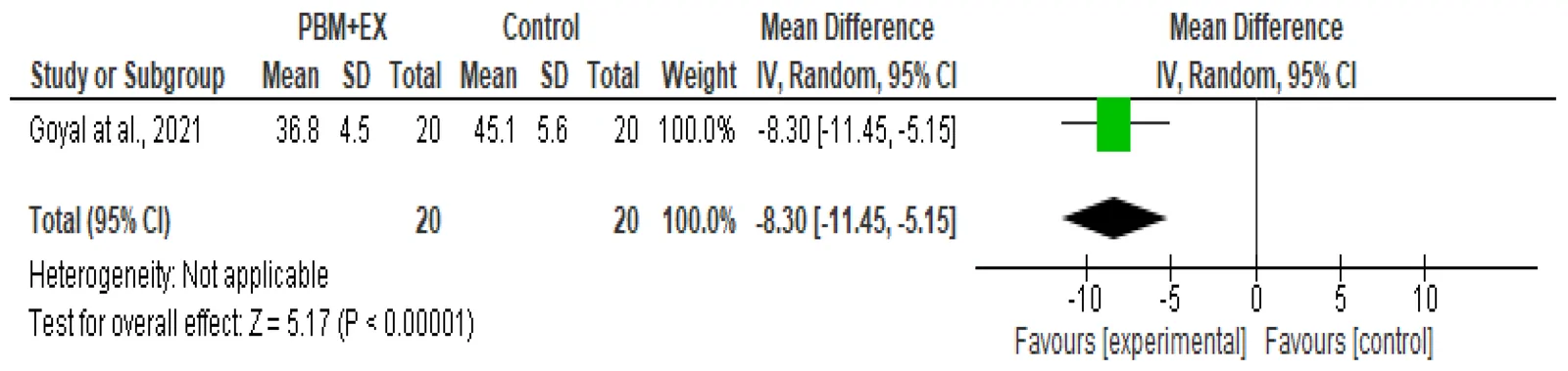
Forest plot of effectiveness of PBM on function (Lower score is better results) [18].

In the initial draft of the manuscript, authors split the meta-analysis into two forest plots. One reflected the effectiveness of PBM on function (Higher score is better results) (Figure 1) and the other showed the effectiveness of PBM on function (Lower score is better results) (Figure 2). Based on a reviewer comment, this multiplication was performed by changing the direction of the measured scale not the value of the measured variable.

However, authors acknowledge that despite transformation, AKPS and WOMAC do not represent identical constructs, and their pooled interpretation may be less precise. In addition, the issue raised regarding potential negative AKPS values in Eurcherdkul et al. [12] is an important observation. It may reflect reporting format (change scores vs. post-treatment values) or data extraction coding decisions.

Authors confirm that all extracted data were checked independently by multiple reviewers. Authors agree that future analyses should consider separating functional outcomes by instrument type (AKPS-only subgroup and WOMAC-only subgroup), which may improve interpretability and reduce indirectness.

## 6. Heterogeneous PBM Modalities and Need for More Homogeneous Subgroup Analysis

The comment emphasized heterogeneity in populations, PBM modalities (LLLT vs. HILT), dosimetry, protocols, and participant characteristics. They suggested subgroup analysis based on PBM modality or protocol.

Authors strongly agree. Heterogeneity in PBM parameters (wavelength, power, energy density, session duration, and total dose) is a well-known limitation in PBM literature and a major contributor to statistical heterogeneity. Furthermore, the inclusion of both low-level and high-intensity laser protocols introduces variability in biological dosing mechanisms and clinical effectiveness.

Authors attempted subgroup analysis based on PBM combined with exercise versus PBM alone, but authors acknowledge that subgrouping based on modality type (LLLT vs. HILT vs. cluster PBM) could provide more mechanistic and clinically useful insights [19,20,21]. Unfortunately, the limited number of RCTs prevented robust subgroup analyses, as many subgroups would include only one or two trials [22].

Authors agree that future evidence updates with larger trial numbers should apply more refined subgroup analyses and potentially dose–response models.

## 7. Need to Update Search Beyond January 2025

The authors recommended extending the search beyond January 2025 due to rapid expansion of PBM literature.

Authors appreciate this recommendation. Our final search was conducted up to January 2025 as reported. Authors agree that PBM research is expanding rapidly and that future updates should include additional trials published after this date. Authors emphasize that systematic reviews represent evidence up to a defined timepoint, and the findings should be interpreted within that scope.

Authors plan to update the review in the future to include newly published RCTs and re-assess the pooled effect estimates and certainty of evidence accordingly.

## 8. Conclusions of Response

In conclusion, authors thank the commentators for their constructive and insightful critique. Authors agree that methodological refinements related to multi-arm trial handling, co-intervention heterogeneity, effect measure selection, functional outcome pooling, and subgroup analyses are important for improving transparency and clinical interpretation. While our review provides preliminary evidence suggesting that PBM may improve pain and function in patellofemoral pain syndrome, authors acknowledge that heterogeneity, methodological limitations of included studies, and limited trial numbers reduce certainty and justify cautious interpretation.

Authors appreciate the opportunity to clarify these points and believe this scholarly discussion strengthens the quality of evidence synthesis in the PBM field.

## Data Availability

Raw data were recorded and maintained in Microsoft Excel by the corresponding author and are available to the editor upon reasonable request. Summarized data relevant to the study findings are presented in the article and/or Supplementary Material of the original manuscript. Further inquiries regarding data availability may be directed to the corresponding author.
